# Clinical and Metabolic Parameters in Non-Small Cell Lung Carcinoma and Colorectal Cancer Patients with and without *KRAS* Mutations

**DOI:** 10.3390/ijerph110908645

**Published:** 2014-08-25

**Authors:** Ahmet Yilmaz, Nehad Mohamed, Kara A. Patterson, Yan Tang, Konstantin Shilo, Miguel A. Villalona-Calero, Michael E. Davis, Xiao-Ping Zhou, Wendy Frankel, Gregory A. Otterson, Weiqiang Zhao

**Affiliations:** 1 Department of Pathology, The Ohio State University, Columbus, OH 43210, USA; E-Mails: Ahmet.Yilmaz2@osumc.edu (A.Y.); Nehad.Mohamed@osumc.edu (N.M.); Kara.Patterson@osumc.edu (K.A.P); Yan.Tang@osumc.edu (Y.T.); Konstantin.Shilo@osumc.edu (K.S.); Xiao-Ping.Zhou@osumc.edu (X.-P.Z.); Wendy.Frankel@osumc.edu (W.F.); 2 Department of Internal Medicine, The Ohio State University, Columbus, OH 43210, USA; E-Mails: Miguel.Villalona@osumc.edu (M.A.V.-C.); Greg.Otterson@osumc.edu (G.A.O.); 3 Department of Animal Sciences, The Ohio State University, Columbus, OH 43210, USA; E-Mail: Davis.28@osu.edu

**Keywords:** *KRAS*, non-small cell lung carcinoma, colorectal cancer, transition, transversion

## Abstract

Lung cancer (LC) and colorectal cancer (CRC) are the first and second deadliest types of cancer worldwide. EGFR-based therapy has been used in the treatment of these cancers with variable success. Presence of mutations in the *KRAS* driver oncogene, possibly induced by environmental factors such as carcinogens in diet and cigarette smoke, may confer worse prognosis and resistance to treatment for reasons not fully understood. Data on possible associations between *KRAS* mutational status and clinical and metabolic parameters, which may help in clinical management, as well as in identifying risk factors for developing these cancers, are limited in the current literature. We sequenced the *KRAS* gene and investigated the associations of variations in 108 patients with non-small cell lung carcinoma (NSCLC), the most common form of LC, and in 116 patients with CRC. All of the mutations originated from the guanosine nucleotide and over half of all transversions in NSCLC and CRC were c.34 G>T and c.35 G>T, respectively. c.35 G>A was the most frequent type of transition in both cancers. Excluding smoking, the clinical and metabolic parameters in patients carrying mutant and wild type *KRAS* were similar except that the CRC patients with transversion mutations were 8.6 years younger than those carrying the transitions (*P* < 0.01). Dyslipidemia, hypertension, family cancer history, and age of diagnosis older than 60 years were more frequent in NSCLC than CRC (*P* ≤ 0.04). These results suggest that most of the clinical and metabolic parameters investigated in this study are probably not associated with the more aggressive phenotype and differences in response to EGFR-based treatment previously reported in patients with *KRAS* mutations. However, the increased rates of abnormal metabolic parameters in patients with NSCLC in comparison to CRC indicate that these parameters may be more important in the management of NSCLC. CRC patients carrying transition mutations are older than those carrying transversions, suggesting that age may determine the type of *KRAS* mutation in CRC patients.

## 1. Introduction

Lung cancer (LC) and colorectal cancer (CRC), the first and second deadliest types of cancer, are responsible for over 22% of all cancer deaths worldwide [1,2]. Mutations in *KRAS*, possibly induced by environmental factors, such as cigarette smoke in LC and diet in CRC, have been reported frequently [3,4]. *KRAS* (v-Ki-ras2 Kirsten rat sarcoma viral oncogene homolog) is a driver oncogene encoding for a small GTPase that activates proteins such as RAF, MEK, and ERK1/2 involved in the MAPK/ERK signal transduction pathway in response to extracellular signals received by the EGFR [5,6]. Mutations in *KRAS* result in the loss of its GTPase activity and constitutive activation of the downstream proteins, changes in G1 and G2/M cell-cycle transit times, decreased apoptosis rates, malignant transformation, and may confer worse prognosis and resistance to EGFR-based treatment [7,8,9,10].

It is known that carcinogens in cigarette smoke and Fe^++^ in red meat directly cause mutations in *KRAS* [11,12]. Smoking and unhealthy dietary habits are also associated with abnormal metabolic parameters such as dyslipidemia, hypertension, and presence of diabetes mellitus either directly due to exposure of cells to mutagens or indirectly via obesity-related hormonal changes [13]. However, possible associations between *KRAS* mutational status and metabolic measurements, which may help in clinical management of patients, as well as in identifying risk factors for developing these cancers, have not been investigated in the literature except that a patient with abnormal metabolic parameters and the *KRAS* G12C mutation has been reported recently [14]. 

Mutations in *KRAS* can be either transitions or transversions. Transitions are generally induced by alkylation, epigenetic events, or oxidative deamination, whereas transversions are generally induced by ionizing radiation or carcinogens such as those found in cigarette smoke. Changes in the normal ratio of transitions to transversions in *KRAS* have been reported in several cancers including subtypes of chronic lymphocytic leukemia and esophageal cancer [15,16]. The changes in the ratio may depend on mutagens exposed or organs involved [17,18]. High rates of transitions have been reported in patients with methylation of the promoter of the DNA repair gene *O^6^-methylguanine-DNA methyltransferase* (*MGMT*) [19]. MGMT specifically repairs G>A transitions and lack of its expression is associated with high rates of transitions [20].

The objective of this study was to investigate possible associations of clinical and metabolic parameters with *KRAS* mutational status in patients with CRC and NSCLC. The nature and type of mutations, as well as transition to transversion ratios, in these patients were also investigated.

## 2. Materials and Methods

DNA was isolated from tissue samples taken after surgery and embedded in paraffin and fixed in formalin. We developed and validated a nested Polymerase Chain Reaction (PCR) assay followed by direct sequencing to identify *KRAS* mutations in codons 12 and 13, the mutational hotspot where over 90% of all *KRAS* mutations have been reported [21]. The forward 5′-TACTGGTGGAGTATTTGATAGTG-3′ and reverse 5′-CTGTATCAAAGAATGGTCCTG-3′ primers were used in the first round of PCR to amplify 5 to 100 ng of DNA in a 25 µL PCR reaction. A second pair of primers was used to amplify one µL of the PCR products obtained in the first round using the forward 5′-TGTAAAACGGCCAGTTAGTGTATTAACCTTATGTG-3′ and reverse 5′-CAGGAAACAGCTATGACCACCTCTATTGTTGGATCATATTCG-3′ primers. 

PCR conditions used in the first round consisted of 95°C for 15 min and 30 cycles of 94°C for 30 sec, 48°C for 30 sec, and 72°C for 30 sec. A final extension step was carried out at 72°C for 7 min. The same conditions were used in the second round of PCR except that the annealing temperature was raised to 58°C. The PCR products were purified using the Qia-Quick PCR purification kit (Qiagen Inc., Valencia, CA, USA) and were subjected to fluorescence-based capillary electrophoresis in an ABI 3130XL genetic analyzer (Life Technologies,Grand Island, NY, USA) to detect the mutations. 

Results were interpreted using the SeqScape v2.6 sequencing analysis software (Life Technologies, Grand Island, NY, USA). The allelic ratio of mutated * versus* wild type alleles was calculated using the formula ((mutant peak height)/(wild type peak height + mutant peak height))× 100. Allelic ratio of 5% or higher was interpreted as the presence of *KRAS* mutation. Thirteen negative and two positive tissue samples previously diagnosed at The Ohio State University Medical Center (Columbus, OH, USA) or the MD Anderson Cancer Center (Houston, TX, USA) were re-tested using the PCR and analysis methods described above for cross-validation and identical results were obtained.

Counts and ratios were analyzed using the nonparametric Mann-Whitney test. Age was analyzed using Student’s *t*-test. Categorical variables were analyzed using the χ^2^ test except that Fisher’s exact test was used if the sample size in any of the cells in a contingency table was less than five. The significance level was set at *P* = 0.05.

## 3. Results

### 3.1. Comparison of the clinical and metabolic parameters in patients with and without KRAS mutations

Smoking was more frequent in NSCLC patients carrying mutated than those carrying the wild type *KRAS* (*P* < 0.001, Table 1). The remaining variables were similar between the patients with and without mutations in *KRAS*. The similar variables included average age at diagnosis, and rates of male gender, age younger than 60 years at the time of diagnosis, alcohol consumption, diabetes, dyslipidemia, hypertension, individual cancer history, and family cancer history. 

### 3.2. Comparison of the clinical and metabolic parameters in patients with different types of KRAS mutations

**Figure 1:**
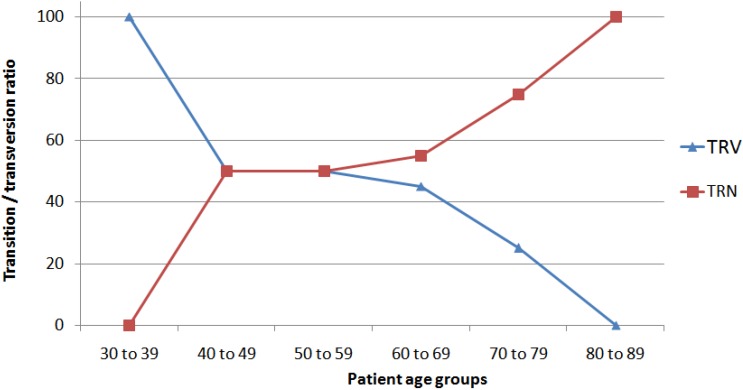
Association of *KRAS* transition/transversion ratios with age groups in patients with colorectal cancer (CRC).

We compared the parameters in patients carrying different types of *KRAS* mutations (*i.e.*, transitions and transversions) to investigate whether these parameters were related to the type of mutations (Table 2). There was no statistically significant difference in any of the variables investigated except that the CRC, but not LC, patients carrying the transition mutations were 8.6 years older than those carrying the transversion mutations (61.03 (11.0) * vs.* 52.4 (10.9) years, *P* < 0.01). To further investigate this finding, we categorized the CRC patients into six different age groups (*i.e.*, 30 to 39 years, 40 to 49 years, and so on). Plotting the transition/transversion ratios against the age groups showed that the transition mutations were absent in the youngest age group (*i.e.*, 30 to 39 years) but were the most frequent type in the oldest age group (*i.e.*, 80 to 89 years) (Figure 1).

### 3.3. Comparison of the clinical and metabolic parameters in NSCLC and CRC patients with the same KRAS mutational status

These comparisons showed that older age at diagnosis, smoking, dyslipidemia, hypertension, and family cancer history were more frequent in NSCLC than in CRC patients when *KRAS* mutations were present (*P* ≤ 0.04, Table 3). Older age at diagnosis and smoking were again more frequent in NSCLC than CRC when no *KRAS* mutations were present.

### 3.4. Comparison of the clinical and metabolic parameters in NSCLC and CRC patients with the same KRAS mutation type

To investigate whether the differences seen in parameters in NSCLC and CRC patients carrying *KRAS* mutations were associated with the type of *KRAS* mutation, we compared the parameters in these patients carrying the same type of *KRAS* mutation (Table 4). These comparisons did not show any significant differences when transitions were present. However, older age at diagnosis, smoking, and dyslipidemia were more frequent in NSCLC than CRC when transversions were present.

### 3.5. Nucleotide and amino acid changes in the NSCLC and CRC patients with KRAS mutations

Changes in the nucleotides and amino acids, as well as the total transition and transversion rates, in patients included in this study are presented in Table 5. c.34 G>T and c.35 G>T were the most frequent transversions in NSCLC and CRC, respectively. c.35 G>A was the most frequent type of transition in both cancers.

**Table 1:** Clinical and metabolic parameters of the 108 non-small cell lung cancer (NSCLC) and 116 colorectal cancer (CRC) patients screened for *KRAS* mutations.

Variables	NLSCS			CRC		
	MUT ^a^	WT ^a^	*P*	MUT ^a^	WT ^a^	*P*
Patients	52	56		56	60	
Average age (SD), years	61.0 (8.9)	62.0 (11.6)	0.34	56.89 (11.7)	56.1 (12.0)	0.36
Male gender	26/51 (51.0)	29/56 (51.8)	0.93	25/56 (44.6)	36/60 (60.0)	0.10
Younger than 60 years of age ^b^	14/32 (43.8)	14/42 (33.3)	0.37	30/56(53.6)	34/60 (56.7)	0.74
Smoker	44/48 (91.7)	36/52 (69.2)	<0.001 *	25/56 (44.6)	25/59 (42.4)	0.81
Consumes alcohol	12/47 (25.5)	7/46 (15.2)	0.23	9/56 (16.1)	12/59 (20.3)	0.56
Diabetic	8/47 (17.0)	7/46 (15.2)	0.82	10/56 (17.9)	9/60 (15.0)	0.69
Dyslipidemia present	20/47 (42.6)	14/46 (30.4)	0.23	9/56 (16.1)	13/60 (21.7)	0.46
Hypertension present	32/48 (66.7)	26/47 (55.3)	0.27	26/56 (46.4)	26/60 (43.3)	0.75
Individual cancer history present	6/47 (12.8)	5/46 (10.9)	0.79	2/56 (3.6)	5/59 (8.5)	0.30
Family cancer history present	33/47 (70.2)	26/46 (56.5)	0.18	28/56 (50.0)	32/60 (53.3)	0.72

Notes: **^a^** Number of patients with each variable. Values in parentheses represent percentages. Abbreviations used: MUT = mutations in *KRAS* present, WT = no mutations in *KRAS* were found. **^b^** Younger than 60 years of age at the time of diagnosis.

**Table 2:** Clinical and metabolic parameters in non-small cell lung carcinoma (NSCLC) and colorectal cancer (CRC) patients with transition or transversion mutations in *KRAS*.

Variables	NSCLC			CRC		
	TRN ^a^	TRV ^a^	*P*	TRN ^a^	TRV ^a^	*P*
Patients	14	38		29	27	
Average age (SD), years	62.1 (9.6)	60.5 (8.6)	0.32	61.03 (11.0)	52.4 (10.9)	<0.01 *
Male gender	10/14 (71.4)	16/37 (43.2)	0.08	12/29 (41.4)	13/27 (48.1)	0.62
Younger than 60 years of age ^b^	6/11 (54.5)	8/21 (38.1)	0.39	13/29 (44.8)	17/27 (63.0)	0.19
Smoker	10/12 (83.3)	34/36 (94.4)	0.28	17/29 (58.6)	13/27 (48.1)	0.45
Consumes alcohol	5/14 (35.7)	7/33 (21.2)	0.32	4/29 (13.8)	5/27 (18.5)	0.64
Diabetic	1/14 (7.1)	7/33 (21.2)	0.27	5/29 (17.2)	5/27 (18.5)	0.91
Dyslipidemia present	7/14 (50.0)	13/33 (39.4)	0.51	6/29 (20.7)	3/27 (11.1)	0.35
Hypertension present	11/14 (78.8)	21/34 (61.8)	0.28	16/29 (55.2)	10/27 (37.0)	0.18
Individual cancer history present	1/14 (7.1)	5/33 (15.1)	0.49	1/29 (3.4)	1/27 (3.7)	0.97
Family cancer history present	11/14 (78.5)	22/33 (66.7)	0.43	16/29 (55.2)	12/27 (44.4)	0.44

Notes: **^a^** Number of patients with each variable. Values in parentheses represent percentages. Abbreviations used: TRN = transition mutation(s) in *KRAS* present, TRV = transversion mutation(s) in *KRAS* present. **^b^** Younger than 60 years of age at the time of diagnosis.

**Table 3:** Clinical and metabolic parameters in non-small cell lung carcinoma (NSCLC) and colorectal cancer (CRC) patients with the same *KRAS* mutational status.

Variables	*KRAS* Mutant			*KRAS* Wild Type		
	NSCLC ^a^	CRC ^a^	*P*	NSCLC ^a^	CRC ^a^	*P*
Patients	52	56		56	60	
Average age (SD), years	61.0 (8.9)	56.89 (11.7)	0.04 *	62.0 (11.6)	56.1 (12.0)	<0.001 *
Male	26/51 (51.0)	25/56 (44.6)	0.53	29/56 (51.8)	36/60 (60.0)	0.38
Younger than 60 years of age ^b^	14/32 (43.8)	30/56 (53.6)	0.40	14/42 (33.3)	34/60 (56.7)	0.02 *
Smoker	44/48 (91.7)	25/56 (44.6)	<0.01 *	36/52 (69.2)	25/59 (42.4)	<0.01 *
Consumes alcohol	12/47 (25.5)	9/56 (16.1)	0.25	7/46 (15.2)	12/59 (20.3)	0.51
Diabetic	8/47 (17.0)	10/56 (17.9)	0.91	7/46 (15.2)	9/60 (15.0)	0.98
Dyslipidemia present	20/47 (42.6)	9/56 (16.1)	<0.01 *	14/46 (30.4)	13/60 (21.7)	0.31
Hypertension present	32/48 (66.7)	26/56 (46.4)	0.04 *	26/47 (55.3)	26/60 (43.3)	0.23
Individual cancer history present	6/47 (12.8)	2/56 (3.6)	0.10	5/46 (10.9)	5/59 (8.5)	0.69
Family cancer history present	33/47 (70.2)	28/56 (50.0)	0.04 *	26/46 (56.5)	32/60 (53.3)	0.76

Notes: **^a^** Number of patients with each variable. Values in parentheses represent percentages. **^b^** Younger than 60 years of age at the time of diagnosis.

**Table 4:** Clinical and metabolic parameters in non-small cell lung carcinoma (NSCLC) and colorectal cancer (CRC) patients with transition or transversion mutations in *KRAS*.

Variables	Transition(s) in *KRAS* Present			Transversion(s) in *KRAS* Present		
	NSCLC ^a^	CRC ^a^	*P*	NSCLC ^a^	CRC ^a^	*P*
Patients	14	29		38	27	
Average age (SD), years	62.1 (9.6)	61.03 (11.0)	0.39	60.5 (8.6)	52.4 (10.9)	<0.001 *
Male gender	10/14 (71.4)	12/29 (41.4)	0.07	16/37 (43.2)	13/27 (48.1)	0.72
Younger than 60 years of age ^b^	6/11 (54.5)	13/29 (44.8)	0.59	8/21 (38.1)	17/27 (63.0)	0.12
Smoker	10/12 (83.3)	17/29 (58.6)	0.15	34/36 (94.4)	13/27 (48.1)	<0.01 *
Consumes alcohol	5/14 (35.7)	4/29 (13.8)	0.13	7/33 (21.2)	5/27 (18.5)	0.81
Diabetic	1/14 (7.1)	5/29 (17.2)	0.40	7/33 (21.2)	5/27 (18.5)	0.81
Dyslipidemia present	7/14 (50.0)	6/29 (20.7)	0.06	13/33 (39.4)	3/27 (11.1)	0.02 *
Hypertension present	11/14 (78.8)	16/29 (55.2)	0.15	21/34 (61.8)	10/27 (37.0)	0.06
Individual cancer history present	1/14 (7.1)	1/29 (3.4)	0.66	5/33 (15.1)	1/27 (3.7)	0.17
Family cancer history present	11/14 (78.5)	16/29 (55.2)	0.15	22/33 (66.7)	12/27 (44.4)	0.09

Notes: ^a^ Number of patients with each variable. Values in parentheses represent percentages. ^b^ Younger than 60 years of age at the time of diagnosis.

**Table 5:** Nucleotide and amino acid changes in *KRAS* in patients with non-small cell lung carcinoma (NSCLC) and colorectal cancer (CRC).

Type of *KRAS* Mutation ^a^	NSCLC ^b^	CRC ^b^	*P*
c.34 G>T transversion (G12C)	22 (57.9)	7 (25.9)	0.04 ^c^
c.34 G>C transversion (G12R)	1 (2.6)	2 (7.4)	
c.35 G>T transversion (G12V)	9 (23.7)	14 (51.9)	
c.35 G>C transversion (G12A)	3 (7.9)	3 (11.1)	
c.37 G>T transversion (G13C)	3 (7.9)	1 (3.7)	
c.34 G>A transition (G12S)	1 (7.1)	4 (13.8)	0.44 ^d^
c.35 G>A transition (G12D)	11 (78.6)	17 (58.6)	
c.38 G>A transition (G13D)	2 (14.3)	8 (27.6)	
Total transversions	38 (73.1)	27 (48.2)	<0.01 *^,e^
Total transitions	14 (26.9)	29 (51.8)	
**TOTAL**	52 (100)	56 (100)	

Notes: **^a^** “c” indicates the number of nucleotide in the cDNA sequence. **^b^** Number of patients with each variable. Values in parentheses represent percentages. **^c^** *P* is the significance value for the χ^2^ test with four degrees of freedom. **^d^** *P* is the significance value for the χ^2^ test with two degrees of freedom. **^e^** *P* is the significance value for the χ^2^ test with one degree of freedom.

Because over half of all transversions in NSCLC and CRC patients consisted of the c.34 G>T and c.35 G>T, respectively, we compared clinical and metabolic parameters in patients with these mutations to those carrying the remaining types of transversions. These analyses did not show any significant differences in the variables analyzed (data not shown).

## 4. Discussion

*KRAS* is a driver oncogene frequently mutated in both NSCLC and CRC [22]. Associations of *KRAS* mutational status with progression, treatment outcome, and response to EGFR-based treatment have been reported in both of these cancers [21,23,24]. Behaviors such as cigarette smoking and consumption of certain food items are known to cause *KRAS* mutations [11,12]. Cigarette smoking and unhealthy diet are additionally associated with abnormal metabolic parameters such as hypertension, dyslipidemia, and presence of diabetes mellitus [13], suggesting that *KRAS* mutational status may be related to metabolic parameters. Such relationships could be important in the treatment as well as clinical management of the patients because the abnormal metabolic parameters themselves are putative risk factors for developing these cancers [25,26].

### 4.1. Comparison of the clinical and metabolic parameters in patients with and without KRAS mutations

Smoking was more frequent in NSCLC patients carrying mutations in *KRAS* (Table 1). All of the remaining parameters were similar between patients with and without mutations, suggesting that *KRAS* mutations were probably not associated with changes in these variables. 

Similar age of diagnosis in patients carrying mutated and the wild type *KRAS* in our study is in agreement with previous studies [27,28]. In our study, *KRAS* mutation frequency in male and female patients was also similar. The role of gender in LC is subject to controversy in the literature. Some authors reported no difference in *KRAS* mutational status between the males and females [29], whereas others reported increased mutations in males [30,31,32] or females [33,34]. These discrepancies may be due to differences in sample size, patient characteristics such as ethnicity, smoking habits, age, or tumor characteristics such as grade and anatomical location. The role of gender in CRC is also subject to controversy in the literature. Some studies reported increased *KRAS* mutations in females [27,35], whereas others reported no difference between the two genders [28]. In a recent large study including a Brazilian cohort of 8234 metastatic CRC patients, *KRAS* mutations were more frequent among females [36].

Increased frequency of *KRAS*-mutated NSCLC among smokers found in our study is in agreement with previous studies [31,33,37]. The role of smoking in CRC has not been well established in the literature, although there is some evidence suggesting that some of the smoking characteristics such as inhalation and smoking frequency may increase CRC risk. Ex-smokers, but not current smokers, may be at increased risk for CRC without any mutation in *KRAS* when compared to those who never smoked [38]. Older women who smoke are also at increased risk for CRC with wild type *KRAS* [39]. Associations between cigarette smoking and a higher risk of developing both hyperplastic and adenomatous polyps with *KRAS* mutations have been reported [40].

Our results on alcohol intake in CRC patients do not agree with a previous study that reported a significant association between alcohol consumption and *KRAS* mutational status [41]. The absence of correlations between *KRAS* mutational status and individual and family cancer history in LC and CRC patients included in our study is in agreement with previous studies [42,43]. Taken together, our results suggest that the worse outcome and resistance to EGFR-based treatment seen in patients carrying mutations in *KRAS* are probably not related to the clinical parameters investigated. 

### 4.2. Comparison of the clinical and metabolic parameters in patients with different types of KRAS mutations

Comparison of the parameters in patients carrying transition and transversion mutations did not show significant differences in the variables investigated except that the CRC patients carrying transition mutations were 8.6 years older than those carrying the transversion mutations (*P* < 0.01, Table 2). To our knowledge, the older age in CRC patients with transition mutations has been reported only by Einspahr *et al.* (2006) [2]. To further investigate this age difference, we categorized patients into six age groups. Interestingly, the transition/transversion ratio was zero at the youngest (*i.e.*, 30 to 39 years) but 100% at the oldest age group (*i.e.*, 80 to 89 years) (Figure 1). Increased transition rates in older CRC patients may be related to epigenetic events or the type or duration of exposure to mutagens in food, although further studies are needed to determine the exact cause of this phenomenon. It is known that *MGMT* is more frequently methylated in the CRC patients older than 60 years of age [20]. Since *MGMT* repairs G>A transitions resulting from formation of guanine adducts in DNA, methylation of its promoter may result in increased transition rates in older CRC patients [44]. Increased rate of transversions in younger patients may be due to high consumption of foods rich in animal fat or potential mutagens [45]. It would be interesting to investigate whether the increased transition rates in older individuals are also present in other genes, especially *p53*, in these as well as other cancer types.

### 4.3. Comparison of the clinical and metabolic parameters in NSCLC and CRC patients with the same KRAS mutational status

These comparisons showed that the NSCLC patients had higher rates of older age at diagnosis, smoking, dyslipidemia, hypertension, and family cancer history than the CRC patients when *KRAS* mutations were present (Table 3). Older age at diagnosis and smoking were again more frequent in NSCLC than in CRC patients who did not carry any mutations in *KRAS*, suggesting that these differences were probably due to reasons other than *KRAS* mutational status. 

EGFR-based treatment may be more effective in NSCLC than in CRC patients carrying wild type *KRAS* [46]. Our results suggest that this difference in treatment outcome could partly be due to the differences in metabolic parameters investigated in our study. Significant family history of cancer in *KRAS*-mutated NSCLC in our study implies that smoking habits may be inherited in families of these patients.

### 4.4. Comparison of the clinical and metabolic parameters in NSCLC and CRC patients with the same KRAS mutation type

None of the clinical and metabolic parameters investigated were different between the NSCLC and CRC patients when transition mutations were present (Table 4). However, older age at diagnosis, smoking, and dyslipidemia were more frequent in NCSCL than in CRC patients when transversion mutations were present. 

Dyslipidemia may increase cancer risk by inducing fatty acid synthase (FASN) activity, which is important in *de novo* fatty acid synthesis in the liver (reviewed by Lee *et al.*, [47]). We are not aware of any studies evaluating associations between *KRAS* mutational status and dyslipidemia in the NSCLC and CRC patients except that an NSCLC patient with dyslipidemia, hypertension, and the *KRAS* G12C mutation has been reported recently [14]. Studies investigating associations between CRC and dyslipidemia without regards to the *KRAS* mutational status are limited in the literature and have produced conflicting results. Some authors have reported higher incidence of CRC in patients with dyslipidemia [48], whereas others reported no difference [49,50]. Yang* et al.* [51] reported that dyslipidemia was associated with improved survival and reduced recurrence in both men and women with CRC. Future larger studies are needed for a precise determination of associations between dyslipidemia and NSCLC and CRC risk.

### 4.5. Nucleotide and amino acid changes in NSCLC and CRC patients with KRAS mutations

Nucleotide and amino acid changes in patients included in this study are shown in Table 5. Our results are in general agreement with a previous study that analyzed 59 NSCLC tumors except that the frequency of the c.35 G>C variant was lower in our study (23.7% *vs.* 5.8% of all *KRAS* mutations) [34]. This discrepancy could be related to patient characteristics such as age or smoking habits. Overall rate of transversions and transitions were increased in NSCLC and CRC, respectively (Table 5), a result in agreement with previous studies [46,52]. The c.34 G>T and c.35 G>T transversions were the most common types of transversions in NSCLC and CRC, respectively. The high transversion rates in NSCLC are probably due to exposure to carcinogens in cigarette smoke that specifically induce transversion mutations [53]. 

The c.35 G>A transition was the most frequent type of transition in both cancers, indicating that it is a highly oncogenic mutational hotspot and may be independent of the type of mutagen exposed or organ involved. Previous studies have shown that it confers worse prognosis in CRC [54], but its importance in the prognosis of LC is less clear. 

Comparison of the clinical and metabolic parameters in carriers of the most common forms of transversions (*i.e.*, c.34 G>T in NSCLC and c.35 G>T in CRC) to those carrying the remaining types of transversions did not show significant differences, indicating that the presence of these mutations was probably not associated with changes in the parameters investigated.

The qualitative and quantitative differences in *KRAS* mutational spectra between the NSCLC and CRC patients in our study are likely due to tobacco-related carcinogenesis in NSCLC. Each *KRAS* mutation subtype detected in our study may affect downstream signaling events differently and represent molecular subtypes with distinct clinicopathologic characteristics. Therefore, biological, clinical, therapeutic, prognostic, and predictive implications of these *KRAS* mutation subtypes should further be investigated in larger patient populations.

## 5. Conclusions

The important results obtained in this study include the following: (1) metabolic parameters are similar in patients with and without *KRAS* mutations, and, therefore, *KRAS* mutational status is probably not associated with changes in the metabolic parameters investigated in this study in either NSCLC or CRC, (2) the older age seen in CRC patients carrying the transition mutations may have important implications in the management of patients with CRC where *KRAS* mutational status is a major determinant of treatment options, and (3) dyslipidemia and hypertension are more frequent in NSCLC than CRC, suggesting that these parameters may be more important in the pathogenesis and management of NSCLC. 
